# Realizing Scalable Nano-SiO_2_-Aerogel-Reinforced Composite Polymer Electrolytes with High Ionic Conductivity via Rheology-Tuning UV Polymerization

**DOI:** 10.3390/molecules28020756

**Published:** 2023-01-12

**Authors:** Mianrui Li, Shengguang Qi, Shulian Li, Li Du

**Affiliations:** Guangdong Provincial Key Laboratory of Fuel Cell Technology, School of Chemistry and Chemical Engineering, South China University of Technology, Guangzhou 510641, China

**Keywords:** composite polymer electrolytes, nano-SiO_2_ aerogel, rheology tuning, lithium metal batteries, UV polymerization

## Abstract

Polymer electrolytes for lithium metal batteries have aroused widespread interest because of their flexibility and excellent processability. However, the low ambient ionic conductivity and conventional fabrication process hinder their large-scale application. Herein, a novel polyethylene-oxide-based composite polymer electrolyte is designed and fabricated by introducing nano-SiO_2_ aerogel as an inorganic filler. The Lewis acid–base interaction between SiO_2_ and anions from Li salts facilitates the dissociation of Li^+^. Moreover, the SiO_2_ interacts with ether oxygen (EO) groups, which weakens the interaction between Li^+^ and EO groups. This synergistic effect produces more free Li^+^ in the electrolyte. Additionally, the facile rheology-tuning UV polymerization method achieves continuous coating and has potential for scalable fabrication. The composite polymer electrolyte exhibits high ambient ionic conductivity (0.68 mS cm^−1^) and mechanical properties (e.g., the elastic modulus of 150 MPa). Stable lithium plating/stripping for 1400 h in Li//Li symmetrical cells at 0.1 mA cm^−2^ is achieved. Furthermore, LiFePO_4_//Li full cells deliver superior discharge capacity (153 mAh g^−1^ at 0.5 C) and cycling stability (with a retention rate of 92.3% at 0.5 C after 250 cycles) at ambient temperature. This work provides a promising strategy for polymer-based lithium metal batteries.

## 1. Introduction

With the ever-increasing demand for efficient energy storage devices, lithium ion batteries (LIBs) have been widely used in modern society [1,2,3]. However, LIBs based on graphite anodes encounter a bottleneck because of their energy density limit. Due to the high theoretical capacity and low electrochemical potential of lithium metal, lithium metal batteries (LMBs) have been considered as promising alternatives [4]. Nevertheless, the organic liquid electrolytes used in LMBs are flammable and volatile, which presents serious safety issues [5]. Compared with organic liquid electrolytes, solid state electrolytes (SSEs) hold great promise in reducing safety risks due to their low flammability and lack of leakage. Moreover, SSEs with satisfactory mechanical properties are capable of suppressing the growth of Li dendrites [6]. 

As one of the important branches in SSEs, solid polymer electrolytes (SPEs) exhibit excellent structure flexibility and processability [7]. Poly (ethylene oxide) (PEO) is the most studied polymer type among numerous SPEs due to the high solvation capacity of the ether groups and chain mobility. However, the low ionic conductivity at room temperature and poor mechanical properties are persistent obstacles to the further applications of PEO-based electrolytes [8,9,10]. To tackle these limitations, various approaches have been conducted with PEO-based electrolytes. Plasticizers are introduced into polymer electrolytes to enhance the ionic conductivity, and the electrolytes obtained are called quasi-solid polymer electrolytes (QPEs) [11,12,13,14]. For example, the introduction of 60% succinonitrile (SN) endows poly (vinylidene fluoride-co-hexafluoropropylene) (PVDF-HFP)/ LiTFSI with a high ionic conductivity of 1 mS cm^−1^ at 0 °C [15]. The PEG/LiSTFSI/EC system shows excellent ionic conductivity of 0.19 mS cm^−1^ at room temperature [16]. The plasticizers promote the dissociation of Li salts due to their plasticity and high polarity. Meanwhile, they increase the amorphous content contributing to chain motion. Hence, QPEs with plasticizers typically exhibit high conductivity. However, this solution is realized at the expense of diminished mechanical strength, which leads to an increased risk of lithium dendrite penetration. Dispersing inorganic filler nanoparticles into PEO-based electrolytes to obtain composite polymer electrolytes is an effective way to enhance ionic conductivity as well as mechanical properties [17,18,19,20]. For example, Cui et al. have developed a composite electrolyte with an interconnected SiO_2_ aerogel backbone [9]. The electrolyte shows an enhanced ionic conductivity of 0.6 mS cm^−1^ at 30 °C and an elastic modulus of up to 430 MPa.

Poly (ethylene glycol) acrylates (PEGAs) belonging to PEO-based electrolytes are widely investigated due to their high solvation ability of Li salts, enhanced electrochemical stability, and low crystallization. The solventless UV polymerization is an efficient and environmentally friendly method to fabricate PEGA electrolytes. Molded casting and the in situ method are dominant in the current reported solventless UV polymerization [16,21,22]. Nevertheless, due to the low viscosity of the electrolyte slurry, it is challenging to obtain the PEGA electrolytes with the desired membrane thickness and shape after casting using the above methods. Moreover, the mentioned methods are noncontinuous and not suitable for mass production. Hence, it is necessary to develop a fabrication method promising continuous casting production at a large scale.

In our previous study, we designed and fabricated a QPE with PEGAs as the polymer matrix, tetramethyl urea (TMU) as the plasticizer, poly(ethylene glycol) diacrylate (PEGDA) as the cross-linker, and polyethylene glycol terephthalate (PET) nonwoven as the supported framework [23]. However, the ionic conductivity (0.18 mS cm^−1^) and mechanical properties (e.g., the elastic modulus of 60 MPa) still need further improvement. Herein, we report a nano-SiO_2_-aerogel-reinforced composite QPE prepared via a facile rheology-tuning UV-initiated polymerization. The rheology-tuning slurry (RTS) possesses a suitable viscosity for continuous coating processes, promising scalable production. Furthermore, the modified QPE shows a high ionic conductivity of 0.68 mS cm^−1^ and an elastic modulus (150 MPa) at room temperature. The Li//Li symmetric cells and LiFePO_4_//Li fullcells exhibit superior electrochemical stability with the SiO_2_-modified QPEs. This work provides new perspectives on how to design and fabricate QPEs for practical lithium metal batteries.

## 2. Results and Discussion

### 2.1. Rheology-Tuning UV Polymerization 

**Figure 1:**
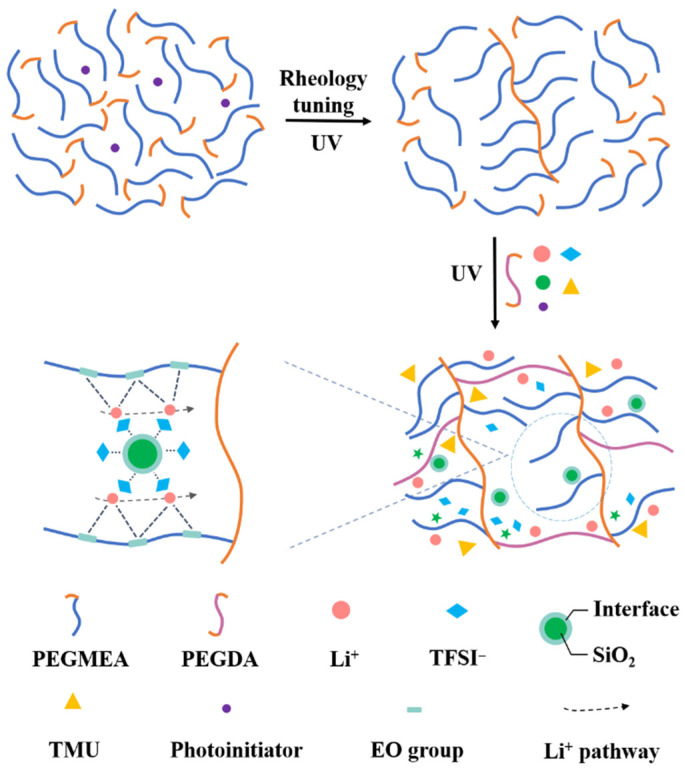
Schematic diagram of the rheology-tuning UV polymerization and the role of SiO_2_.

In this work, rheology tuning is achieved by adjusting the viscosity and thixotropy via UV polymerization. Figure 1 illustrates the mechanism of rheology-tuning polymerization. First, poly(ethyleneglycol) methyl ether acrylate (PEGMEA) monomers are mixed with initiators, and the mixture is exposed to a UV lamp for several minutes until it turns into a viscous slurry. In this step, part of the monomers undergoes C=C bond polymerization. The RTS with polymers as solute and unreacted PEGMEA monomers as solvent belongs to a typical non-Newtonian fluid, possessing the shear-thinning characteristic. The viscosity of the RTS decreases along with the increasing shear rate, proving its shear-thinning behavior (Figure S1). In contrast, the PEGMEA monomers lacking rheology tuning show no shear-thinning behavior and quite low viscosities. Therefore, the RTS is suitable for a continuous coating process. After rheology tuning, the RTS is mixed with PEGDA, Li salts, plasticizer, nano-SiO_2_ aerogel, and photoinitiator, coated on both sides of PET nonwoven, and cured under the UV lamp. The thickness of the electrolyte can be changed by adjusting the gap of the scraper.

To optimize the SiO_2_ content, electrolyte membranes containing 0 wt%, 3 wt%, and 5 wt% SiO_2_ are prepared and tested for their cycling stabilities in Li//Li symmetric cells at 0.1 mA cm^−2^ and 0.1 mAh cm^−2^. When the content of SiO_2_ increases to more than 5 wt%, the electrolyte slurry becomes too viscous to be mixed up evenly. Hence, 5 wt% is selected as the highest content of SiO_2_ in experimental electrolytes. Figure S2 illustrates that the Li//Li cells employing electrolytes with 0 wt%, 3 wt%, and 5 wt% SiO_2_ exhibit a similar polarization voltage of approximately 70 mV. Nevertheless, QPEs with 0 wt% and 3 wt% SiO_2_ exhibit short circuits within only 600 h and 750 h, respectively. By contrast, RTS-5% SiO_2_ QPE holds stable cycling for 750 h, which indicates that RTS-5% SiO_2_ QPE provides the highest mechanical strength to resist Li dendritic penetration in our exploration. Therefore, 5 wt% is selected as the optimized content of SiO_2_ in the following research.

### 2.2. Physicochemical Characterization 

**Figure 2:**
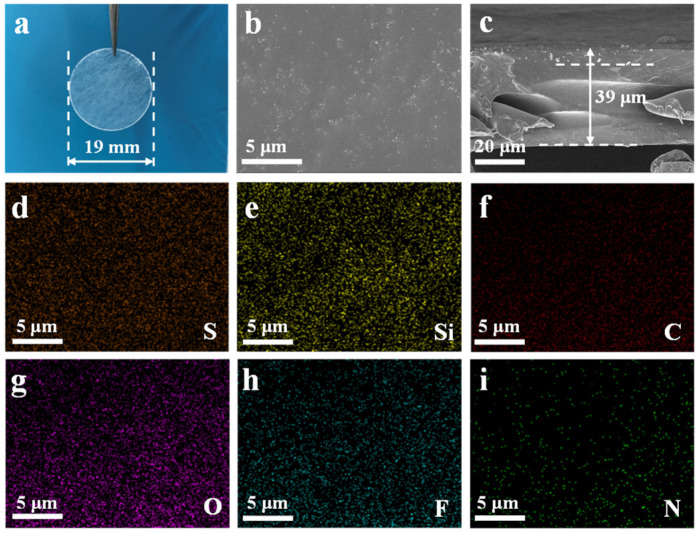
Physical characterization of RTS-5% SiO_2_ QPE. (**a**) Digital photograph of the QPE. (**b**) SEM image of the QPE surface. (**c**) Cross-section SEM image of the QPE. (**d**–**i**) EDS mapping images of S, Si, C, O, F, and N elements in the QPE.

The digital photo of the RTS-5% SiO_2_ QPE membrane is presented in Figure 2a. The RTS-5% SiO_2_ QPE membrane exhibits a flat and smooth surface. As illustrated in Figure 2b, a compact surface without any cracks is obtained on the QPE film. The SiO_2_ is distributed uniformly, and no obvious agglomeration can be observed. Figure 2c shows the cross section of QPE with a thickness of 39 μm. The RTS-5% SiO_2_ QPE slurry is supported by the PET nonwoven and cures successfully. Furthermore, the EDS mapping images are shown in Figure 2d–i. The S, Si, C, O, F, and N elements of RTS-5% SiO_2_ QPE are distributed evenly, which further suggests that the QPE slurry has been coated on PET nonwoven uniformly and compactly.

**Figure 3:**
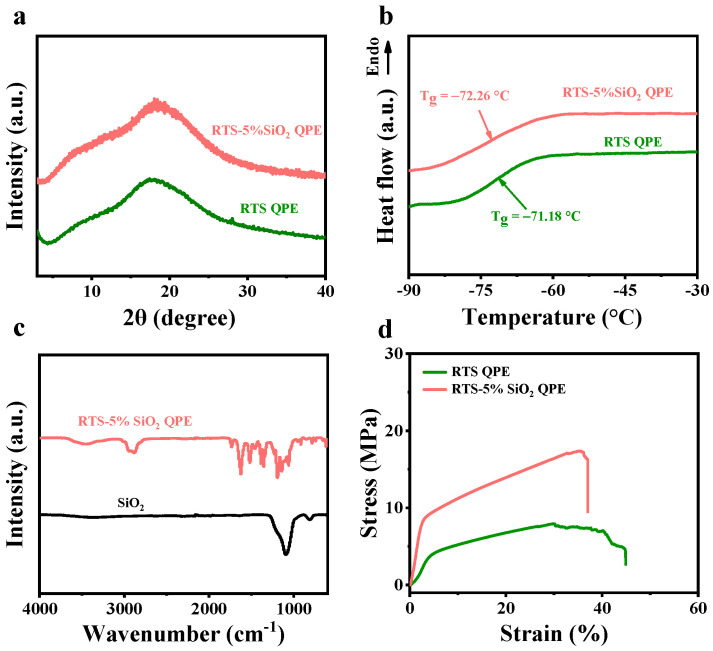
Physical characterization of RTS QPE and RTS-5% SiO_2_ QPE. (**a**) XRD spectra; (**b**) DSC curves; (**c**) FTIR spectra; (**d**) Stress–strain curves.

The X-ray diffraction (XRD) patterns of RTS QPE and RTS-5% SiO_2_ QPE membranes are exhibited in Figure 3a. No crystal state peaks can be observed in the XRD results. The amorphous state leads to improved ionic conductivity of the electrolyte. The electrolytes are also characterized by Differential Scanning Calorimetry (DSC). The T_g_ values of RTS-5% SiO_2_ QPE and RTS QPE are −72.26 °C and −71.18 °C, respectively. It indicates that the electrolytes are amorphous at room temperature, which contributes to the mobility of polymer chains. Fourier transform infrared (FTIR) spectroscopy demonstrates that the characteristic peaks of SiO_2_ exist in RTS-5% SiO_2_ QPE, indicating that the original structure of SiO_2_ remains after being mixed with the polymer slurry. There are no chemical changes that can be observed (Figure 3c).

For polymer electrolytes, satisfactory mechanical properties are essential indices for practical application in batteries. The stress–strain curve results in Figure 3d demonstrate that the RTS-5% SiO_2_ QPE exhibits higher tensile strength (17.38 MPa) than the RTS QPE (7.29 MPa). Moreover, the detailed mechanical properties (Table 1) indicate that the addition of SiO_2_ enhances the tensile strength, maximum load, and elastic modulus, which contributes to suppressing the growth of Li dendrites and increasing the lifetime of cells.

### 2.3. Electrochemical Behaviors of RTS-5% SiO_2_ QPE

**Figure 4:**
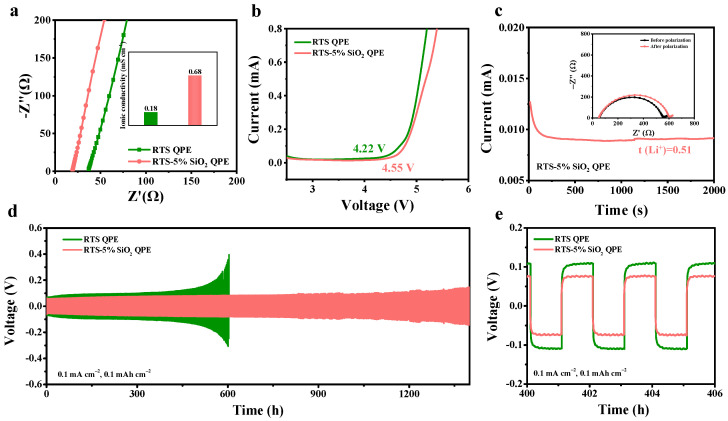
Electrochemical performance of RTS QPE and RTS-5% SiO_2_ QPE. (**a**) Nyquist curves of RTS QPE and RTS-5% SiO_2_ QPE at room temperature. The embedded histogram shows ionic conductivities; (**b**) Linear sweep voltammetry (LSV) curves of RTS QPE and RTS-5% SiO_2_ QPE. The voltage ranges from 2.0 V to 6.0 V at 10 mV s^−1^. (**c**) Current and EIS curves of a Li/RTS-5% SiO_2_ QPE/Li battery before and after polarization. (**d**) The galvanostatic charge–discharge cycling curves of Li//Li symmetric cells sandwiched with RTS QPE and RTS-5% SiO_2_ QPE at 0.1 mA cm^−2^ and 0.1 mAh cm^−2^. (**e**) Zoom-in curves at 400−406 h in (**d**).

To evaluate the Li^+^ transference ability in QPE, the electrochemical impedance spectra are conducted at room temperature (Figure 4a). The RTS-5% SiO_2_ QPE exhibits a higher conductivity (0.68 mS cm^−1^) than the RTS QPE (0.18 mS cm^−1^) does. The increased ionic conductivity can be attributed to the role of the nano-SiO_2_ aerogel. Firstly, the Lewis-acidic sites on SiO_2_ interact with EO groups, which decreases the polymer crystallinity and weakens the interaction between Li^+^ and EO groups [24,25,26]. Second, the Lewis acid–base interaction between the SiO_2_ and TFSI^-^ from Li salts promotes the dissociation of Li salts [27,28]. This synergistic effect produces more free Li^+^ in QPEs. As illustrated in Figure 4b, RTS-5% SiO_2_ QPE is electrochemically stable up to 4.55 V, which is higher than RTS QPE (4.22 V) at room temperature. It demonstrates that SiO_2_ is beneficial for improving the electrochemical stability of QPE. The Li^+^ transference number of RTS-5% SiO_2_ is around 0.51, higher than that of RTS QPE (t (Li^+^) = 0.27) (Figure S3). This is due to an increase in free Li^+^ and a decrease in free TFSI^-^ anions in RTS-5% SiO_2_ QPE. 

To confirm the compatibility of the electrolyte with lithium metal, Li//Li symmetric cells sandwiched with RTS QPE and RTS-5% SiO_2_ QPE are tested at 0.1 mA cm^−2^, with a fixed capacity of 0.1 mAh cm^−2^ (Figure 4d). The overpotential of Li/RTS QPE/Li cells suddenly increases after 600 h of cycling, indicating that the conduction of lithium ions in the electrolyte is blocked. The Li/RTS-5% SiO_2_ QPE/Li, on the other hand, can cycle stably for 1400 h without short circuiting, indicating that the interface between the Li metal and electrolyte is stable during Li deposition and stripping. There are no lithium-ion-conductive obstructions or lithium-dendrite-piercing phenomena. The enlarged curves at 400−406 h demonstrate that the overpotential of Li/RTS-5% SiO_2_ QPE/Li cells is lower than that of RTS-SiO_2_ QPE (Figure 4e), which can be attributed to the increased ionic conductivity of the electrolyte.

### 2.4. Electrochemical Performance of RTS-5%-SiO_2_-QPE-Based FullCells

**Figure 5:**
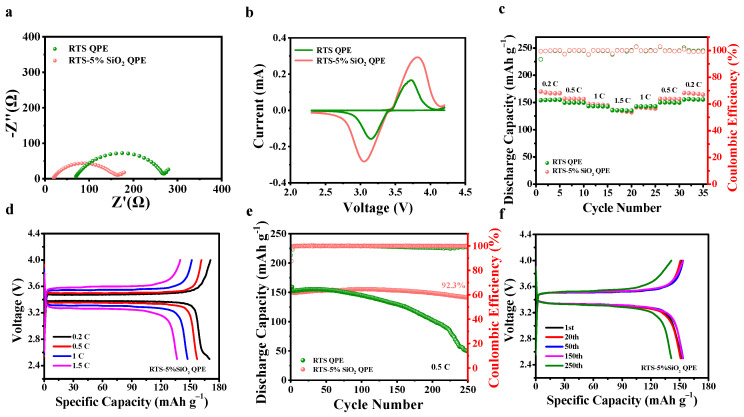
Electrochemical performance of LiFePO_4_/RTS QPE/Li and LiFePO_4_/RTS-5% SiO_2_ QPE/Li at room temperature. (**a**) Nyquist curves of the fullcells employing LiFePO_4_ and Li. (**b**) CV curves of the fullcells. (**c**) Rate performance of fullcells. Galvanostatic charge/discharge curves of the fullcells cycling at (**d**) different rates from 0.2 C to 1.5 C and (**f**) various cycles at 0.5 C. (**e**) Cycling performance and coulombic efficiency of the fullcells in the voltage range of 2.5 to 4.0 V at 0.5 C.

To verify the practical application of the QPEs, LiFePO_4_/QPE/Li fullcells are assembled and tested at ambient temperature. As illustrated in Figure 5a, the RTS-5% SiO_2_ QPE exhibits a lower electrochemical impedance (≈163 Ω) than the RTS QPE (≈270 Ω), implying that the fast ion transference is conducted in the RTS-5% SiO_2_ QPE. Meanwhile, it proves the higher ionic conductivity of RTS-5% SiO_2_ QPE than that of RTS QPE. Cyclic voltammetry measurements are conducted to evaluate the electrochemical redox kinetics. The redox peak intensities of RTS-5% SiO_2_ QPE are higher than those of RTS QPE, indicating the former QPE delivers a higher charge/discharge capacity (Figure 5b). It corresponds to the higher initial discharge capacity of LiFePO_4_/RTS-5% SiO_2_ QPE /Li cells at 1 C (147.5 mAh g^−1^, Figure S4). The rate capability of fullcells is evaluated at various C-rates from 0.2 C to 1.5 C (Figure 5c). The LiFePO_4_/RTS-5% SiO_2_/Li cells deliver specific discharge capacities of 168.3, 157.3, 147.5, and 136.6 mAh g^−1^ at 0.2, 0.5, 1, and 1.5 C. Moreover, the discharge capacity returns to the original values along with the return of current densities. Compared with the RTS-QPE-based batteries, the RTS-5% SiO_2_ QPE shows an excellent rate performance and reversibility. The charge/discharge curves at various C-rates are presented in Figure 5d. In contrast to RTS-QPE-based cells, the RTS-5%-SiO_2_-QPE-based cells exhibit higher specific capacities and lower polarization voltages.

To further test the electrochemical cyclic stability at high current density, galvanostatic charge/discharge cycling measurements are conducted at 0.5 C. After 250 cycles, the full cells employing RTS-5% SiO_2_ QPE exhibit a higher specific capacity (140.7 mAh g^−1^) and capacity retention (92.3%) than RTS QPE (Figure 5e), indicating excellent cycling stability and good interfacial contact between the electrode and RTS-5% SiO_2_ QPE. The galvanostatic charge/discharge curves of the RTS-5%-SiO_2_-QPE-based cells at the first, twentieth, fiftieth, and one hundred fiftieth cycles are presented in Figure 5f. During the first cycle to the one hundredth cycle, the charge/discharge curves almost overlap, and there is no obvious capacity decay, which further proves the superior cycle stability of RTS-5% SiO_2_ QPE. In general, LiFePO_4_/RTS-5% SiO_2_ QPE/Li cells exhibit excellent capacity and electrochemical stability. The comparison for the electrochemical performance of polymer solid state electrolytes is shown in Table S1.

## 3. Materials and Methods

### 3.1. Preparation of RTS Recipe Slurry

First, 50 g of PEGMEA (M_w_ = 518; Sartomer CD551) and 0.02 g of 2,2-dimethoxy-2-phenylacetophenone photoinitiator were added into a flask filled with nitrogen. The mixture was stirred for 10 min to obtain a uniform solution. The solution was then placed under a 365 nm UV lamp for several minutes until a viscous slurry formed. In the meantime, the UV lamp and nitrogen were removed to stop the reaction. Second, 2 g of rheology-tuning slurry and 1 g of PEGDA (M_W_ = 608; Sartomer SR610) were added into a brown glass bottle for preliminary mixing, and then 3 g of TMU, 2 g of LiTFSI, and 0.01 g of photoinitiator were added in turn and stirred to obtain the RTS recipe slurry. 

### 3.2. Preparation of SiO_2_-Modified Quasi-Solid Polymer Electrolyte Membrane

#### 3.2.1. RTS-5% SiO_2_ Recipe Slurry

A total of 0.1 g of nano-SiO_2_ aerogel was added into 2 g of RTS recipe slurry and stirred continuously until the SiO_2_ was completely dispersed. After that, 0.01 g of photoinitiator was added and stirred.

#### 3.2.2. RTS-5% SiO_2_ QPE

RTS-5% SiO_2_ recipe slurry was cast coated on both sides of PET nonwoven and then cured under a 365 nm UV lamp for several minutes to obtain the uniform RTS-5% SiO_2_ QPEs.

The obtained electrolytes are named RTS-*x*% SiO_2_ QPEs (*x* for the mass percentage of SiO_2_ in the electrolytes, detailed in Table S2), while those without SiO_2_ are labeled as RTS QPEs.

### 3.3. Preparation of LiFePO_4_ Cathode

The LiFePO_4_ cathode was fabricated by coating the slurry consisting of LFP (active material, 80 wt%), PVDF (binder, 6 wt%), KS-6 (conductive agent, 2 wt%), Super P (2 wt%), and RTS QPE (10 wt%) on the carbon-coated Al foil (C-Al) and then drying at 60 °C overnight. The C-Al was punched into pellets with a diameter of 8 mm. The mass loading of the cathode obtained was 2.16 mg cm^−2^.

### 3.4. Physical Characterization

A Carl Zeiss SEM was conducted to observe the morphologies of the QPE. The Rigaku MiniFlex (Rigaku) X-ray diffractometer (XRD) was used for the crystal phase test. The test was performed under the following conditions: Cu Kα ray, voltage 35 kV, current 30 mA, scanning step diameter 0.01 s^−1^, and scanning rate 5° min^−1^. The scanning range of the sample was 3−80°. Fourier transform infrared (FTIR) spectra using Nicolet-IS50 (Thermo Fisher Scientific) were utilized to investigate the structure. Differential Scanning Calorimetry (DSC) was conducted on the Polyma 214 instrument. The mechanical properties were evaluated using the Instron 5967 tensile test machine.

### 3.5. Electrochemical Characterization

Electrochemical impedance spectroscopy (EIS) was used to evaluate the ionic conductivity of the QPE. The test was performed on Autolab Electrochemical Instrumentation (Metrohm) in the frequency range of 1 Hz to 100 kHz. The C-Al/QPE/C-Al batteries were assembled for the test. The ionic conductivity (σ) was calculated by the following equation:

$$\sigma =\frac{L}{\mathrm{RA}}$$

where L represents the thickness of the QPE membrane, R is the bulk resistance obtained from alternating current impedance analysis, and A is the contact area between C-Al and the electrolyte.

Li^+^ transference number (t_Li_^+^) was obtained in Li/electrolyte/Li cells at room temperature by combining DC polarization and AC impedance via the following equation:

$$t_{{\mathrm{Li}}^{+}}=\frac{I_{\mathrm{ap}}\left(\Delta V-{\;I}_{\mathrm{bp}}R_{\mathrm{bp}}\right)}{I_{\mathrm{bp}}\left(\Delta V-{\;I}_{\mathrm{ap}}R_{\mathrm{ap}}\right)}$$

where ΔV is the voltage pulse at DC polarization applied to the symmetric cells, and its value is 10 mV. I_bp_ and I_ap_ represent the initial and steady currents, respectively. R_bp_ and R_ap_ represent the resistance before and after polarization, respectively. The linear sweep voltammetry was implemented in Li/QPE/stainless steel (SS) cells at a sweep rate of 10 mV s^−1^ and a voltage range of 2.0 V to 6.0 V. The cyclic voltammetry (CV) was conducted at 0.1 mV s^−1^ in a voltage range of 2.3–4.2 V. To obtain Li symmetric cells, QPE was sandwiched with two Li foils (diameter = 10 mm) in CR2016-type coin cells. To do the full cell assembly, a CR2016-type coin cell was assembled by contacting, in sequence, the LFP electrode (d = 8 mm), the QPE membrane (d = 19 mm), and a lithium foil (d = 10 mm). All batteries were assembled in an argon-filled glove box (H_2_O < 0.1 ppm, O_2_ < 0.1 ppm). On a LAND-CT3001A battery tester (Wuhan LAND Electronic Co., Ltd.), galvanostatic charge/discharge measurements were conducted to test the cyclic performance of Li symmetrical cells and fullcells LiFePO_4_/QPE/Li. The LiFePO_4_/QPE/Li fullcells were tested at a voltage range of 2.5 V−4.0 V. The electrochemical measurement tests described above were all conducted at 27 °C. 

## 4. Conclusions

In summary, a nano-SiO_2_-aerogel-modified QPE is prepared via a rheology-tuning UV-initiated polymerization. The RTS-5% SiO_2_ QPE possesses a high ionic conductivity of 0.68 mS cm^−1^ and mechanical strength at room temperature. The Lewis acid sites on SiO_2_ aerogel interact with EO groups and anions from Li salts, which decreases the polymer crystallinity, boosts the number of free Li^+^, and thus promotes the ionic conductivity. The SiO_2_-aerogel-reinforced electrolyte can not only suppress Li dendrites, but also contribute to the stability of electrochemical cycling. The Li//Li symmetric cells cycle stably over 1400 h. LiFePO_4_/RTS-5% SiO_2_/Li cells deliver impressive cycling stability, with a discharge capacity of 140.7 mAh g^−1^ and 92.3% retention after 250 cycles at 0.5 C. UV-initiated solventless polymerization holds promise for large-scale continuous coating. This work gives new insights into the design and fabrication of composite polymer electrolytes for lithium metal batteries. 

## Figures and Tables

**Table 1:** Mechanical properties of polymer electrolyte membranes.

	RTS QPE	RTS-5% SiO_2_ QPE
Tensile strength (MPa)	7.29	17.38
Breaking elongation (%)	40.93	37.03
Maximum load (MPa)	9.01	23.47
Elastic modulus (MPa)	60	150

## Data Availability

The data presented in this study are available on request from the corresponding authors.

## Supplementary Materials

The following supporting information can be downloaded at: https://www.mdpi.com/article/10.3390/molecules28020756/s1, Figure S1: Viscosity curves of PEGMEA monomer and RTS; Figure S2: (**a**) Galvanostatic charge-discharge curves of Li//Li symmetric cells with RTS-*x*% SiO_2_ QPE (*x* = 0, 3, and 5) at 0.1 mA cm^−2^ and 0.1 mAh cm^−2^; (**b**) Zoom-in curves of 400−406 hours; Figure S3: Chronoamperometry polarization curve as well as the impedance spectra before and after polarization of Li/RTS QPE/Li cell; Figure S4: Initial galvanostatic charge/discharge curves of LiFePO_4_//Li cells at 1 C. Table S1: The comparison for the electrochemical performance of polymer solid state electrolytes; Table S2: Detailed information of RTS-*x*% SiO_2_ QPE (*x* = 0, 3, and 5). References [29,30,31,32,33,34,35] are cited in the supplementary materials.
