# Supplementary material for: Realizing Scalable Nano-SiO2-Aerogel-Reinforced Composite Polymer Electrolytes with High Ionic Conductivity via Rheology-Tuning UV Polymerization
Source: Molecules. 2023 Jan 12;28(2):756. doi: 10.3390/molecules28020756 (PMC9861509; doi:10.3390/molecules28020756)
Supplement: Supplementary file 1 [file molecules-28-00756-s001.zip › molecules-2092199-supplementary.pdf]

Supplementary Materials

Realizing Scalable Nano-SiO_2-Aerogel-Reinforced Composite Polymer Electrolytes with High Ionic Conductivity via Rheology Tuning UV Polymerization

Mianrui Li [†], Shengguang Qi [†], Shulian Li and Li Du *

Guangdong Provincial Key Laboratory of Fuel Cell Technology, School of Chemistry and Chemical Engineering, South China University of Technology, Guangzhou 510641, People's Republic of China;
202120123902@mail.scut.edu.cn (M.L.); samuel_qsg@aliyun.com (S.Q.);
shilling07324@163.com (S.L.);
* Correspondence: duli@scut.edu.cn
[†] These authors contributed equally to this work.

Table S1. The comparison for the electrochemical performance of polymer solid state electrolytes.

**Polymer solid state electrolyte**	**Fabrication method**	**Facile Scalable Yes(Y) or No(N)**	**Temp. (℃)**	**σ (mS cm⁻¹)**	**Current density /cycle time (Li//Li symmetric cell)**	**Retention rate /Cycle number/ C rate ($LiFePO_4$//Li cell)**	**Ref.**
PEG/LiTFSI/SiO_2	In-situ polymerization-induced self-assembly	N	26	0.17	0.1 mA cm^{-2}/600 h	68.1%/300/0.2 C	[1]
PCL/LiTFSI/Al_2O_3	Solution casting	Y	60	0.05	0.1 mA cm^{-2}/500 h	81.3%/500/1.0 C	[2]
PEO/LiTFSI/MnO_2	Solution casting	Y	30	0.02	0.1 mA cm^{-2}/800 h	86.7%/300/0.5 C (60℃)	[3]
PEG/LiTFSI/PILs	In-situ thermal polymerization	N	25	0.10	0.1 mA cm^{-2}/2400 h	93.8%/150/0.2 C	[4]
PEO/LiTFSI/IL	Solution casting	Y	40	0.66	0.1 mA cm^{-2}/1000 h	99.3%/200/1 C	[5]
PEG/LiMTFSI/PC	In-situ thermal polymerization	N	25	0.1	not mentioned	98%/100/0.1 C	[6]
PEG/LiTFSI/LLZTO	Solution casting	Y	20	0.22	0.5 mA cm^{-2}/125 h	88%/150/0.1 C	[7]
PEG/LiTFSI/SiO_2	Rheology tunning UV polymerization	Y	27	0.68	0.1 mA cm^{-2}/1400 h	92.3%/250/0.5 C	This work

Table S2. Detailed information of RTS-*x*% SiO_2 QPE (*x*=0, 3, and 5)

Quasi-polymer electrolytes	PEGMEA (g)	TMU (g)	PEGDA (g)	651 (g)	LiTFSI (g)	SiO_2 (g)	LiTFSI (wt%)	SiO_2 (wt%)
RTS-0% SiO_2	0.4	0.75	0.24	0.01	0.5	0	26.32	0
RTS-3% SiO_2	0.4	0.75	0.24	0.01	0.5	0.06	25.51	3
RTS-5% SiO_2	0.4	0.75	0.24	0.01	0.5	0.1	25	5

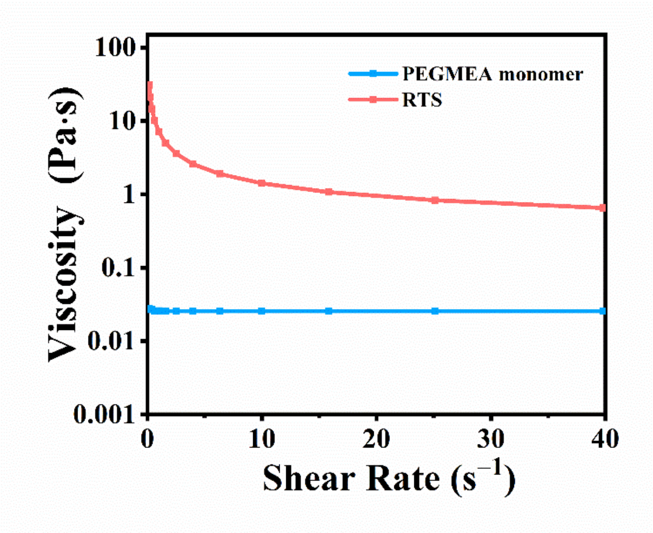

Figure S1. Viscosity curves of PEGMEA monomer and RTS.

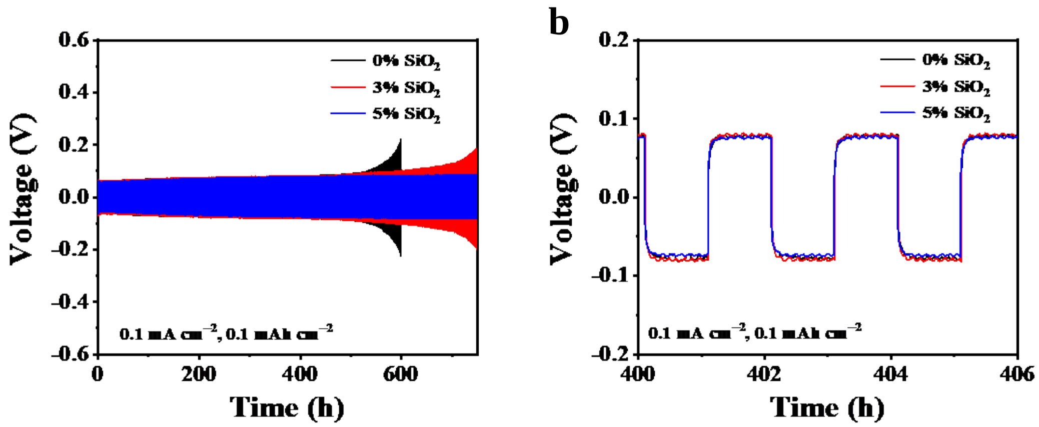

Figure S2. (**a**) Galvanostatic charge-discharge curves of Li//Li symmetric cells with RTS-x% SiO_2 QPE (x=0, 3, and 5) at 0.1 mA cm^{-2} and 0.1 mAh cm^{-2}; (**b**) Zoom-in curves of 400–406 hours.

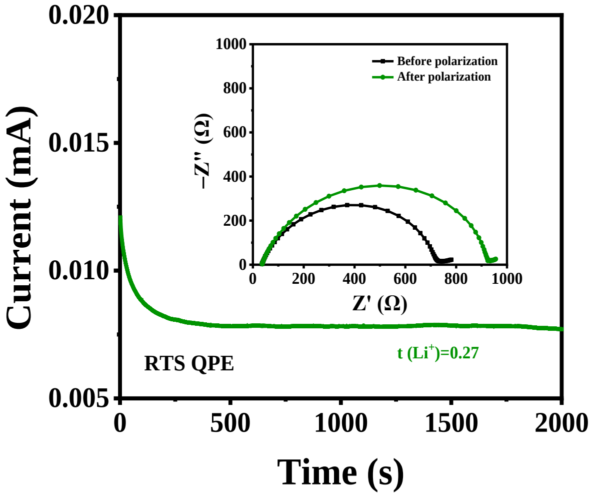

Figure S3. Chronoamperometry polarization curve as well as the impedance spectra before and after polarization of Li/RTS QPE/Li cell.

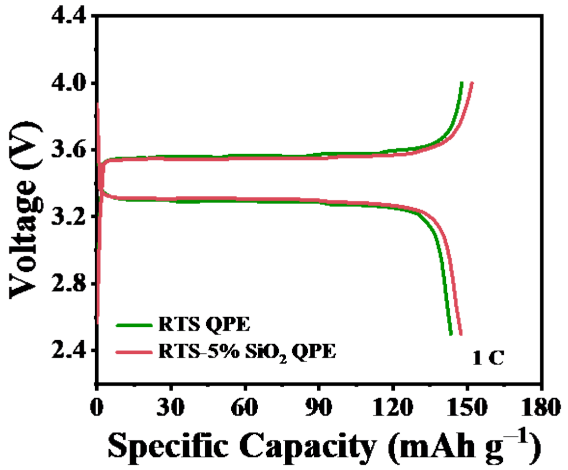

Figure S4. Initial galvanostatic charge/discharge curves of $LiFePO_4$//Li cells at 1 C.

References

1. Yuan, B.; Luo, G.; Liang, J.; Cheng, F.; Zhang, W.; Chen, J., Self-assembly synthesis of solid polymer electrolyte with carbonate terminated poly(ethylene glycol) matrix and its application for solid state lithium battery. Journal of Energy Chemistry 2019, 38, 55-59.
2. Pei, D.; Ma, R.; Yang, G.; Li, Y.; Huang, C.; Liu, Z.; Huang, S.; Cao, G.; Jin, H., Enhanced ion transport behaviors in composite polymer electrolyte: the case of a looser chain folding structure. Journal of Materials Chemistry A 2022, 10, (6), 3226-3232.
3. Li, Y.; Sun, Z.; Liu, D.; Gao, Y.; Wang, Y.; Bu, H.; Li, M.; Zhang, Y.; Gao, G.; Ding, S., A composite solid polymer electrolyte incorporating MnO_2 nanosheets with reinforced mechanical properties and electrochemical stability for lithium metal batteries. Journal of Materials Chemistry A 2020, 8, (4), 2021-2032.
4. Tseng, Y.-C.; Hsiang, S.-H.; Tsao, C.-H.; Teng, H.; Hou, S.-S.; Jan, J.-S., In situ formation of polymer electrolytes using a dicationic imidazolium cross-linker for high-performance lithium ion batteries. Journal of Materials Chemistry A 2021, 9, (9), 5796-5806.
5. Atik, J.; Diddens, D.; Thienenkamp, J. H.; Brunklaus, G.; Winter, M.; Paillard, E., Cation-Assisted Lithium-Ion Transport for High-Performance PEO-based Ternary Solid Polymer Electrolytes. Angewandte Chemie International Edition 2021, 60, (21), 11919-11927.
6. Porcarelli, L.; Shaplov, A. S.; Bella, F.; Nair, J. R.; Mecerreyes, D.; Gerbaldi, C., Single-Ion Conducting Polymer Electrolytes for Lithium Metal Polymer Batteries that Operate at Ambient Temperature. ACS Energy Letters 2016, 1, (4), 678-682.
7. Huo, H.; Zhao, N.; Sun, J.; Du, F.; Li, Y.; Guo, X., Composite electrolytes of polyethylene oxides/garnets interfacially wetted by ionic liquid for room-temperature solid-state lithium battery. Journal of Power Sources 2017, 372, 1-7.
